# Loss of haptic feedback impairs control of hand posture: a study in chronically deafferented individuals when grasping and lifting objects

**DOI:** 10.1007/s00221-019-05583-2

**Published:** 2019-06-17

**Authors:** R. Chris Miall, Orna Rosenthal, Kristin Ørstavik, Jonathan D. Cole, Fabrice R. Sarlegna

**Affiliations:** 1 School of Psychology, University of Birmingham, Birmingham, B15 2TT UK; 2 Department of Neurology, Oslo University Hospital, Oslo, Norway; 3 Centre of Postgraduate Research and Education, Bournemouth University, Bournemouth, UK; 4 Aix Marseille University, CNRS, ISM, Marseille, France

**Keywords:** Human, Proprioception, Somatosensation, Grasp, Haptics, Manipulation

## Abstract

Previous work has highlighted the role of haptic feedback for manual dexterity, in particular for the control of precision grip forces between the index finger and thumb. It is unclear how fine motor skills involving more than just two digits might be affected, especially given that loss of sensation from the hand affects many neurological patients, and impacts on everyday actions. To assess the functional consequences of haptic deficits on multi-digit grasp of objects, we studied the ability of three rare individuals with permanent large-fibre sensory loss involving the entire upper limb. All three reported difficulties in everyday manual actions (ABILHAND questionnaire). Their performance in a reach–grasp–lift task was compared to that of healthy controls. Twenty objects of varying shape, mass, opacity and compliance were used. In the reach-to-grasp phase, we found slower movement, larger grip aperture and less dynamic modulation of grip aperture in deafferented participants compared to controls. Hand posture during the lift phase also differed; deafferented participants often adopted hand postures that may have facilitated visual guidance, and/or reduced control complexity. For example, they would extend fingers that were not in contact with the object, or fold these fingers into the palm of the hand. Variability in hand postures was increased in deafferented participants, particularly for smaller objects. Our findings provide new insights into how the complex control required for whole hand actions is compromised by loss of haptic feedback, whose contribution is, thus, highlighted.

## Introduction

When healthy, we may take for granted our ability to hold everyday objects easily and securely, with minimal attention required to keep the object in hand during movement. We are normally unaware of the complex control issues required to reach and grasp the object, adopting in advance a hand posture shaped to the object itself. Nor are we aware of the changing forces required to securely grip and lift the object, when grip force is precisely coordinated with the voluntary lift to compensate for load and rotational forces (for a review, Johansson and Flanagan 2008). The complexity of such tasks becomes evident only when we detect errors, such as a slip, or complete failure, for example, if our fingers are too cold to provide good sensory information (Cheung et al. 2003), or after a stroke (Nowak et al. 2003) or after numbing the skin over the fingertips (Johansson and Westling 1984; Witney et al. 2004).

In the field of robotics, grasp and object manipulation are recognised as significant challenges (Suárez-Ruiz and Pham 2016). Despite enormous technical advances (see review by Yousef et al. 2011), robots are seriously limited by lack of proprioception (the sense of position and movement of body segments), of touch, and force feedback (Soter et al. 2018). Some pundits even suggest that mundane tasks like picking up and moving small objects will remain for humans for quite some time, while robots can take over other apparently complex tasks (computation, planning, driving, medical diagnosis and the like; Manyika et al. 2017; D’Anna et al. 2017). On the other hand, a challenge remains for bio-engineers interested in developing prostheses, for instance, for amputees who complain about the lack of haptic feedback necessary for everyday actions with upper-limb prostheses (Hochberg et al. 2006; Aflalo et al. 2015; D’Anna et al. 2017). In this article, we refer to grasp as the fixed hand posture used to hold an object (Feix et al. 2016), manipulation as handling or control of an object (i.e. with changing hand postures) and haptic feedback (Grafton 2010) as sensory inputs arising during the interaction with objects, from touch and proprioception.

Human grasp of objects is highly stereotypical (Reilmann et al. 2001; Castiello 2005). A number of classification schemes have been proposed that capture this finite range of postures [see Schlesinger 1919, Taylor and Schwartz 1955 cited in Cutkosky (1989); Napier 1956; Kamakura et al. 1980; Kapandji 1989; Cutkosky 1989; Cesari and Newell 2000]. These taxonomies have recently been rationalised into a single classification of 33 grasp postures (Feix et al. 2016). One example of a mechanically simple grasp is achieved by a precision grip, a pinch using just index finger and thumb, which can be behaviourally characterised by the grip aperture (distance between thumb and index fingers) and by grip force. Precision grip aperture has been studied extensively in reach-and-grasp tasks, and experiments with individuals with haptic loss have revealed the importance of sensory feedback for accurate control of aperture during the reach towards the object (Jeannerod et al. 1984; Jeannerod 1986; Gentilucci et al. 1994; Simoneau et al. 1999; Jackson et al. 2000).

Grip and load forces between the thumb and one (or all 4) fingers are also accurately controlled as the object is moved (Danion and Sarlegna 2007; Johansson and Flanagan 2008; Hermsdörfer et al. 2008). Again, the impact of haptic loss is well documented, for instance, when the finger pads are numb after anaesthesia, grip force is excessive and coordination is poor (Westling and Johansson 1984; Augurelle et al. 2003; Monzee et al. 2003). Excessive grip force and impaired coordination have also been reported in patients deprived of haptic feedback because of a sensory neuropathy (Gentilucci et al. 1994; Hermsdörfer et al. 2008; Thonnard et al. 1997).

Using more than two digits can provide greater stability than the precision grip between thumb and index finger (Napier 1956; Cutkosky 1989; Saudabayev et al. 2018), albeit at the cost of controlling more muscles and joints. Studies have shown that haptic loss impairs handwriting and manipulating small objects (Gentilucci et al. 1994; Rothwell et al. 1982; Hepp-Reymond et al. 2009; Danna and Velay 2017). A patient with a severe haptic loss was found to perform a grooved pegboard test in ~ 14 min (Cuadra et al. 2019), which is ten times longer than controls (Ruff and Parker 1993). Augurelle et al. (2003) reported that 7/10 participants dropped an object during anaesthesia of the fingerpads, while none would normally drop it. Power grip of larger objects is less affected (but see Enders and Seo 2017). Based on such work (see also Shibata and Santello 2017), one might expect that the range and variation of multi-digit hand postures would be altered after the loss of haptic feedback. However, to the best of our knowledge, there has been no systematic study of the effects of reduced haptic sensation on the hand postures adopted during object grasp.

We had the opportunity to assess the grasping behaviour of three people who had each suffered a chronic sensory neuronopathy, between 15 and 40 years prior to our tests, that led to the permanent loss of large-diameter sensory afferents from below the neck. Such deafferented individuals are effectively deprived of all the tactile and proprioceptive signals normally used in haptics. However, their motor pathways are intact and muscle power is normal. Considering their massive sensory deficit, they are remarkably adept when using visual supervision to control upper-limb movements (Miall et al. 2018). For two of these three individuals, many other aspects of their sensorimotor control have previously been reported, including reaching movements with and without vision (Ghez et al. 1995; Hoellinger et al. 2017), their ability to adapt to novel visual or dynamic perturbations (Ingram et al. 2000; Sarlegna et al. 2010b; Lefumat et al. 2016), and their judgements about the weight of objects (Miall et al. 2000; Fleury et al. 1995). The third participant has not taken part in many studies, but we recently described her sensory loss and her abilities in motor control, perceptual judgements, and in motor learning (Miall et al. 2018).

Chronically deafferented participants offer a model of what might be possible in terms of control of multi-digit hand movement in the face of profound sensory loss. The study was specifically designed to assess the posture of the hand and digits when holding various objects in a steady and secure grasp, with four hypotheses. On previous occasions, one of these three participants, IW, has spoken of his strategies to simplify grasping actions by mainly using thumb and index fingers or thumb, index and middle fingers, especially in the early years when he was relearning to control his body after the neuronopathy. This strategy would be consistent with his reduction of degrees of freedom observed when he was able to walk (Lajoie et al. 1996). Thus, our first hypothesis was that deafferented participants would use simplified postures, compared to controls, that reduce their grasp to 2 or 3 digits when possible (Reilmann et al. 2001). IW has also spoken of the difficulty in controlling the fingers when visual feedback is lost—for example, when the fingers are occluded by the object he is grasping, suggesting that there may be a benefit of manipulating transparent objects at least for deafferented individuals. Thus, second, we expected differential grasping of transparent vs opaque objects, because of the effects of visual occlusion and possible compensatory visuomotor mechanisms developed by deafferented individuals. Based on previous work showing the impact of deafferentation on upper-limb movements (Gordon et al. 1995; Ghez et al. 1995; Sarlegna et al. 2006, 2010b), we thirdly hypothesised a greater variability of performance in deafferented individuals compared to neurologically intact participants. Finally, given their loss of haptic feedback and potentially excessive grip forces, we expected reduced inter-finger distances when holding compliant objects compared to control participants.

## Methods

### Participants

#### Deafferented participants

Three participants (IW, GL and WL) who live with a chronic, stable sensory neuronopathy participated in these experiments. Details of their neurology are reported in (Miall et al. 2018; see also Cole and Paillard 1995); in summary, all three experienced a specific, massive loss of large, myelinated sensory fibres as adults that for IW and GL occurred 35–40 years before testing, and 15 years for WL. They have normal motor pathways as assessed by conduction velocity, and normal muscle innervation, assessed by EMG, but have no sensation of touch in the arms or hand, no sense of position and movement of the unseen fingers, hands and arms, and no stretch reflexes in the limbs. They have clinically normal thermosensation and nociception. They were each tested during a two-day visit to Birmingham University, in a single test session held between other experiments that are reported elsewhere (Miall et al. 2018). IW is male, 61 years old at the time of participation, 100% left-handed according to the 10-item version of the Edinburgh inventory (Oldfield 1971) and became deafferented at age 19; GL is female, 66 years old at the time of participation, right-handed with a laterality quotient of 77% (Lefumat et al. 2016) and became deafferented at age 31; WL is also female, 46 years old at the time of participation, 100% left-handed according to the 10-item version of (Oldfield 1971). She also became deafferented at age 31 and was then immobilised for ~ 2 months before gradually improving over a 2-year period; she has remained ataxic in her arm and hand movement, unlike IW, who does not have arm ataxia, while GL has modest hand/arm ataxia. (Ataxia is characterised by impaired coordination, gait and postural imbalance; characteristic arm movements are poorly controlled in speed, force and direction, and often include ‘hunting’ around the target position). With their hands fully spread, the thumb–little finger distance was 18, 23 and 20 cm for GL, IW and WL, respectively.

#### Control participants

Six control participants were recruited. The age range was 51–67 (mean 60.3 years) and three were females. All but one (female) were self-reported right-handed; all used their preferred, dominant hand in the experiment.

#### Hand function

All participants were asked to answer the ABILHAND questionnaire to assess manual function in adults with neuromuscular disorders (Vandervelde et al. 2010), rating the perceived difficulty of 18 everyday activities on a three-point scale (impossible, difficult or easy). The results of the analysis (performed on http://www.rehab-scales.org) are reported in Table 2.

#### Ethics

The University of Birmingham STEM ethics committee approved all experiments. All participants were provided with written and verbal information about the task prior to the experiment, and gave their written consent, according to the Declaration of Helsinki.

### Experimental set-up

Participants were seated comfortably in front of a wooden desk. The deafferented participants used their own wheelchairs; controls sat on a standard office chair. Each participant used their preferred hand, and the 3D position of each digit was recorded using the Polhemus Fastrak motion tracker with miniature sensors (12 × 10 × 20 mm) taped to the dorsum of each distal phalanx. 3M Micropore surgical tape provided a thin, slip-free surface to the dorsum and sides of the fingertips; the fingerpad was free of tape. A sixth sensor was taped in the midline of the dorsum of the hand; two additional sensors were attached to the object to be grasped and to the table surface as a fixed reference position, respectively. Before the recording session began, the position of each motion tracker was individually calibrated to provide the estimated offset from the marker to the fingertip or to the palm of the hand, respectively (see “Data analysis”, below).

Twenty objects were chosen to cover a range of shapes, sizes, weights and materials, and all details are reported in Table 1. The list included plastic, glass or wooden rigid cylindrical tubes, a wooden pencil, rigid plastic sheets, balls of various sizes, and blocks of rubber foam of different densities. Each object was in turn instrumented with one of the Polhemus sensors, except the two smallest objects (a marble and a small rubber ball) as the presence of a sensor would have compromised the grasping action. Each object was then placed on the table in front of the participant, at a comfortable reaching distance (approximately 20 cm from the edge of the table). Most objects were placed directly on the table; narrow tubes and the plastic sheets were held vertically by an experimenter, and released immediately after grasp by the participant. The smallest balls were placed on a paper towel, to avoid them rolling on the table-top. Once the object was in place, the participant was asked to reach out at a normal speed, to grasp and lift it about 10–20 cm off the table, and then return it onto the table and release it. Participants did not interact with the objects before recording but they were able to see them placed onto the table, and they were presumably familiar with these classes of everyday materials. Reaches were performed in blocks of five trials per object, i.e. each object was lifted five times in succession. In this initial study, we did not restrict the starting position or posture of the hand, which, therefore, varied to some extent between participants, and also between objects.

**Table 1:** Details of the objects used

Object	Class	Material	Transparent	Rigidity	Mass (g)	Length (mm)	Width (mm)	Height (mm)	Diameter (mm)
1	Pencil	Wood	N	H	5	180	–	–	7
2	Rigid sheets	Perspex/plexiglass	Y	H	40	150	2	150	–
3		Black plastic	N	H	40	150	2	150	–
4	Cylinders	White plastic	N	H	15	–	–	180	15
5		Perspex/plexiglass	Y	H	45	–	–	200	15
6		White plastic	N	H	45	–	–	180	40
7		Glass	Y	H	110	–	–	110	40
8		Plastic pipe insulation	N	M	5	–	–	180	45
9		Black plastic	N	H	120	–	–	180	70
10		Glass	Y	H	160	–	–	120	70
11		Plastic pipe insulation	N	M	20	–	–	180	75
12		High-density cardboard	N	H	320	–	–	200	120
13	Spheres	Glass	N	H	5	–	–	–	15
14		Rubber	N	M	15	–	–	–	30
15		Rubber/felt (tennis ball)	N	M	60	–	–	–	68
16		Rubber	N	M	225	–	–	–	120
17	Cuboids	Soft foam	N	VL	30	100	50	100	–
18		Firm foam	N	L	20	100	50	100	–
19		Extra firm foam	N	M	40	100	50	100	–
20		Rigid foam	N	H	18	100	50	100	–

The objects were all common, from either household or hardware. The sheets and cylinders were selected to include transparent (Y = Yes) and opaque versions (N). Rigidity of the objects is given from very low, low, medium to high (VL, L, M, H). Mass is reported to the nearest 5 g and exclude the mass of the motion tracking sensor and the proximal section of its cable (approx. 20 g)

### Data analysis

The motion tracking sensors were sampled at 240 Hz and analysed off-line with Matlab scripts. The main outcome of the data processing pipeline was a record of the relative positions of the five fingertips and the palm of the hand in three dimensions at the moment of maximal elevation.

#### Calibration

Prior to testing, and after the motion trackers were taped in place, the position of each sensor on the dorsum of the fingers or hand was calibrated with respect to the finger pad (the usual contact point between the fingers and objects) or the centre of the palm, respectively. The participant was requested to place the fingertip or palm on a fixed wooden disk with a central raised conical section, providing a blunt pivot about 1 cm high around which the finger could be rotated with visual guidance. They then rotated their hand and fingers to maximise movement of the sensor around the fixed pivot provided by the fingertip/palm on the wooden cone. For the deafferented participants, this process was passive, with an experimenter placing the tip of a pencil-like wooden stick that carried a position tracker at its far end against the participant’s fingertip, rotating it while holding the finger stable (in other words, the fixed fingertip provided the pivot around which the marker was rotated). A Matlab script then minimised the variance of the estimated fingertip position with respect to the moving sensor, using the *minqdef* quadratic minimization function, to find the 3D offset between the sensor and the pivot point. This was repeated for all sensors in turn and visually inspected to ensure a good calibration and reconstruction of a single position (we typically recovered a spread of estimated pivot positions of less than 1–2 mm). We will from now on refer to these extrapolated positions on the finger pads and on the palm of the hand as the “finger” or “hand” positions.

#### Hold postures during object lifting

To extract the finger-hold positions from each trial, the height of the sensor attached to the object was measured and its maximal vertical position noted. For the two smallest objects (a marble and a small rubber ball), the average vertical position of all six markers on the hand and fingers was used instead, and the moment of maximum elevation noted. Next, the trajectories of these six markers were visually examined, with the moment of the maximum object/hand average lift position marked, to ensure correct identification of a moment when the object was held aloft. The three-dimensional data on finger and hand position from each of the five trials with the same object were then aligned by applying a rigid spatial transformation and rotation to best align the sets of six markers from each trial to the mean of all five trials using the same object (using the Matlab *procrustes* function with scaling and reflection blocked). This process aligns the data from each trial, regardless of the absolute position or orientation of the hand but, importantly, the absolute distances between the six markers (the five fingers and the palm) are unchanged by the *procrustes* process, such that hold data of all five trials per object can be used for further analysis. Again, visual inspection of the cluster of positions ensured correct identification of the hold positions. Any marker position that was found to be more than five standard deviations from the mean of the position of that marker on the other four trials was then excluded (causes include occasional data communication errors and sometimes one trial very different from the others: for IW and GL, we had to exclude the data from 1 or 2 fingers for one object, respectively). Finally, the distances between all pairs of the six markers were computed, and the average and standard deviation of these distances calculated for each block of five trials for each of the twenty objects. Finally, the distance matrix was scaled to lie between 0 and 1 for each participant, thus normalising the data for different hand sizes. This analysis was designed to reduce the data from the six markers to a very limited number of metrics, so that comparison across trials, objects and participants was possible. Through an oversight, the small glass cylinder (object 5) was not tested for IW and WL.

#### Reach-to-grasp trajectories

As a secondary aspect of the analysis, we also measured the reach trajectory towards each object, but report detailed data for only one object, the high-density foam cuboid (object 20) chosen as an exemplar. We captured the speed of hand motion from the start to the moment the object was moved, from the marker on the hand. We also measured the aperture of the precision grip (the distance between the thumb and index fingers) throughout the reach-to-grasp action. Reach onset was taken as the moment hand speed exceeded 5% of the maximum for that trial; reach offset was the moment of minimum speed before the first upward movement of the hand and object. Mean trajectories were calculated across the five trials to each object, after resampling the time-course data from each trial to 100 time points, i.e. into percentage of reach duration. Reach data were unavailable for participant WL as we only sampled the lift, hold and replacement of each object on that occasion.

#### Statistics

Because of the small group, we generally contrast the three participants individually against the control group by calculation of *t* scores:

$$t\, = \,(x_{\text{individual}} - {\text{group}}_{\text{mean}} )\,/\,[{\text{group}}_{{{\text{SD}}}}\,/\,{\text{sqrt}}\left( {{\text{group}}_{N} } \right)],$$

highlighting instances where *t* > 3 (*p* = ~ 0.01). We also use mixed-model ANOVAs, with factors of group (control vs deafferented) and object, in SPSS.

## Results

### Questionnaire of manual ability for adults with neuromuscular disorders

The list of tasks in the ABILHAND questionnaire can be ordered by their difficulty (Vandervelde et al. 2010). While a healthy individual is supposed to judge each of the 18 everyday tasks on this list as easy, only the first two tasks were judged as easy by all three deafferented participants and WL and GL reported that the eight most difficult tasks were either difficult or impossible (Table 2). In contrast, IW reported that only two tasks were difficult. On later questioning, he said that he has adopted many strategies that allow most of these activities to be performed easily, but would find doing them “the normal way” difficult or impossible.

**Table 2:**
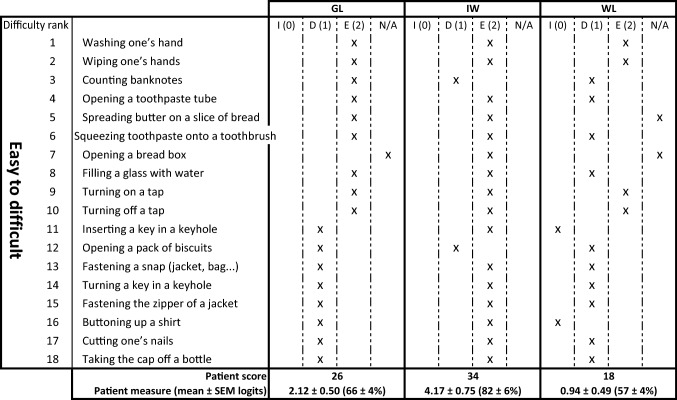
Results of the ABILHAND questionnaire for adults with neuromuscular disorders

Tasks are judged as Impossible (I, score 0), difficult (D, score 1), Easy (E, score 2) or N/A if not attempted within 3 months. Healthy adults find all 18 tasks easy, and score 36. The patient measure (mean ± SEM) is linear measure of manual ability, calibrated on a scale (approx. ± 6.2 logits) established for patients with neuromuscular disorders (Vandervelde et al. 2010)

### Kinematic analysis of reach–grasp–lift movements

All control participants found the task straightforward, despite the presence of the sensors on the fingers, except when grasping the two smallest diameter objects (the pencil and the marble, objects 1 and 13). Here, the sensors on the back of the distal finger segments sometimes would come into contact with the table-top surface. However, typically after one trial, participants adjusted their hand orientation and finger positions during the approach phase, and their remaining lifts were achieved successfully. Some also found the largest 12-cm-diameter cylinder too big to grasp with one hand, due to the size of their hands, as did participant GL; they, therefore, used a bimanual action for this object (but we report only the data from the dominant hand). The deafferented participants found lifting the smallest items more difficult and were noticeably slower than controls for these trials, consistent with previous reports (Gentilucci et al. 1994; Hoellinger et al. 2017). Deafferented participants also found lifting the pencil from the surface of the table into a writing posture difficult and used unusual strategies. GL and WL used the other hand to help stabilise the pencil, which was then moved into a unimanual writing position; IW pressed on the pencil tip with his other index finger to lift the pencil shaft off the table, which he then grasped with his dominant hand.

#### *Reach*-*to*-*grasp trajectories*

**Fig. 1:**
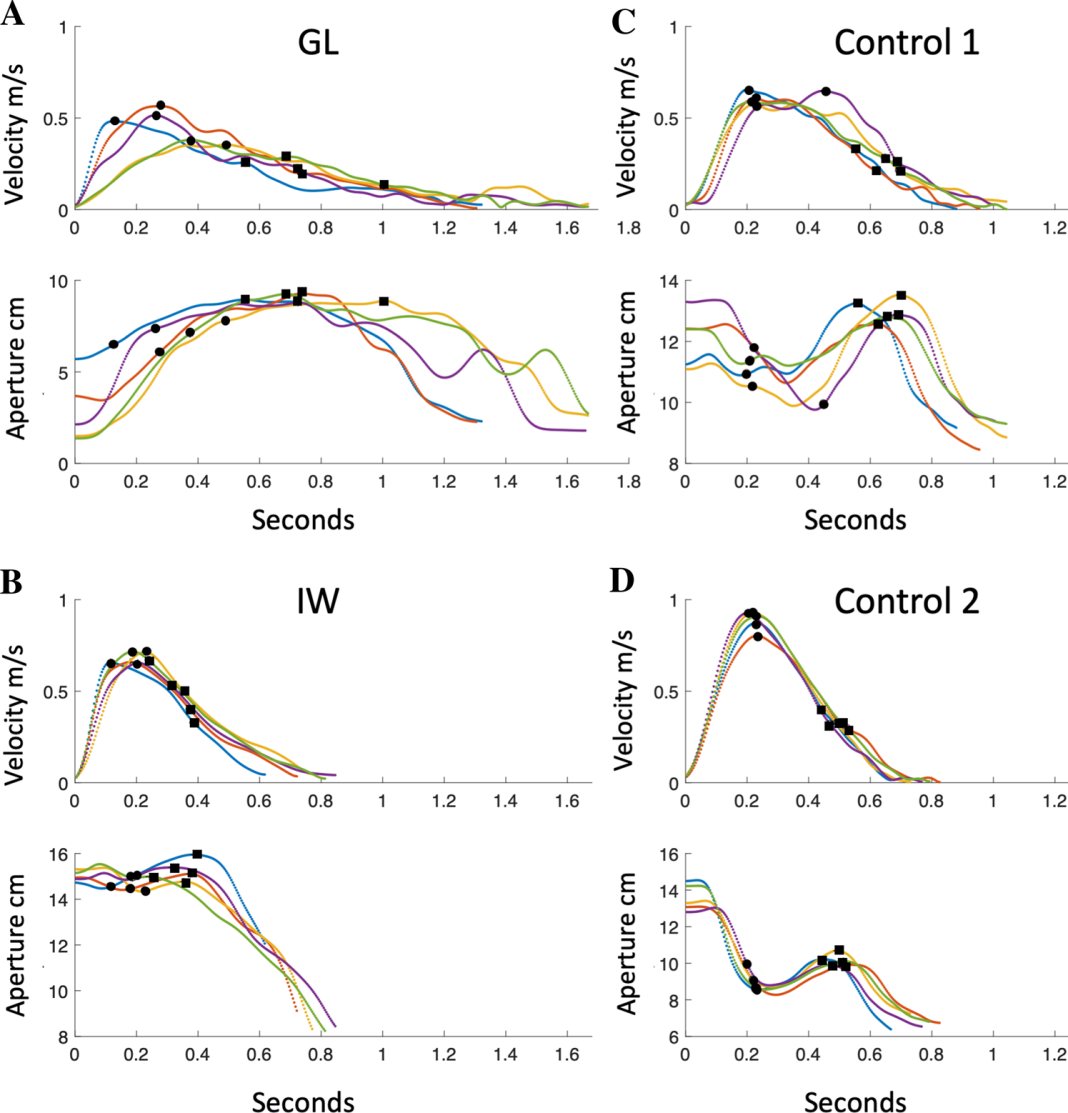
Examples of reach-to-grasp behaviour. **a**–**d** Data from five trials reaching and lifting the rigid foam cuboid (object 20), for two deafferented participants (**a**, **b**) and two controls (**c**, **d**). The upper graph in each panel shows the reach speed profile as measured by the marker attached to the back of the hand; the lower graphs show the grip aperture between thumb and index finger. The moments of peak speed and the subsequent moments of maximum aperture are indicated with dots and squares, respectively

Although our main goal was to study hand postures during object holding, kinematic analysis of the hand reach trajectories was possible for two of the deafferented participants, GL and IW. Because reach distance was not constrained, we do not provide detailed statistical analyses. Figure 1 shows the time course of exemplar reaches by GL and IW to the rigid foam cuboid, the last object picked up by each participant. We also compare their reaches with two control participants, selected to have the most similar speed profiles to GL and IW overall. IW and GL appeared slower than these selected controls, as reflected by the lower peak speed of the reach-to-grasp phase, and GL in particular showed a long ‘tail’ to her speed profiles. IW’s movement duration was comparable to the example control shown in Fig. 1d, but the distance travelled was shorter (Fig. 3b vs d), and hence a slower average speed. To assess whether the relative speed at the grasp–closure phase was lower in GL and IW compared to the control group, we compared the hand speed at 75% of movement duration, during the closure phase. A mixed-model ANOVA (group vs objects) reported significant differences for the within-participant factor of object [*F*(18,108) = 2.1, *p* = 0.009], but no group effect, and no interaction. Hence, while they tended to approach more slowly than controls, reach distance would need to be constrained to possibly confirm this.

Figure 1 also shows that the controls dynamically changed aperture during the second half of the reach: in these two examples, aperture reduced from the initial high starting point, before they scaled opening and closing onto the object during the final approach. In contrast, both GL and IW tended to open their grip early (synchronous with or soon after peak speed) and then maintained this open posture throughout the approach before the final closure phase (with considerable trial-to-trial variability for GL). To assess this, we normalised aperture to its maximum in each trial, and calculated the average from the moment of peak speed until the end of the reach trajectory. A mixed-model ANOVA reported a significant group effect [*F*(1,6) = 9.7, *p* = 0.021], an object effect [*F*(18,108) = 8.5, *p* < 0.001], but no significant interaction. GL and IW appeared to adopt a greater safety margin in their aperture (mean across objects: 0.87 and 0.92, respectively) than controls (grand mean across subjects and objects 0.80). We calculated *t* scores to compare separately GL and IW to the controls, for each object. For 19 of the objects, both GL and IW had higher average normalised aperture: *t* > 3 for 10/20 objects for GL (objects 2, 5, 7, 8, 10 and 16–20; see Table 1) and 16/19 objects for IW (all but objects 6, 8, 11).

**Fig. 2:**
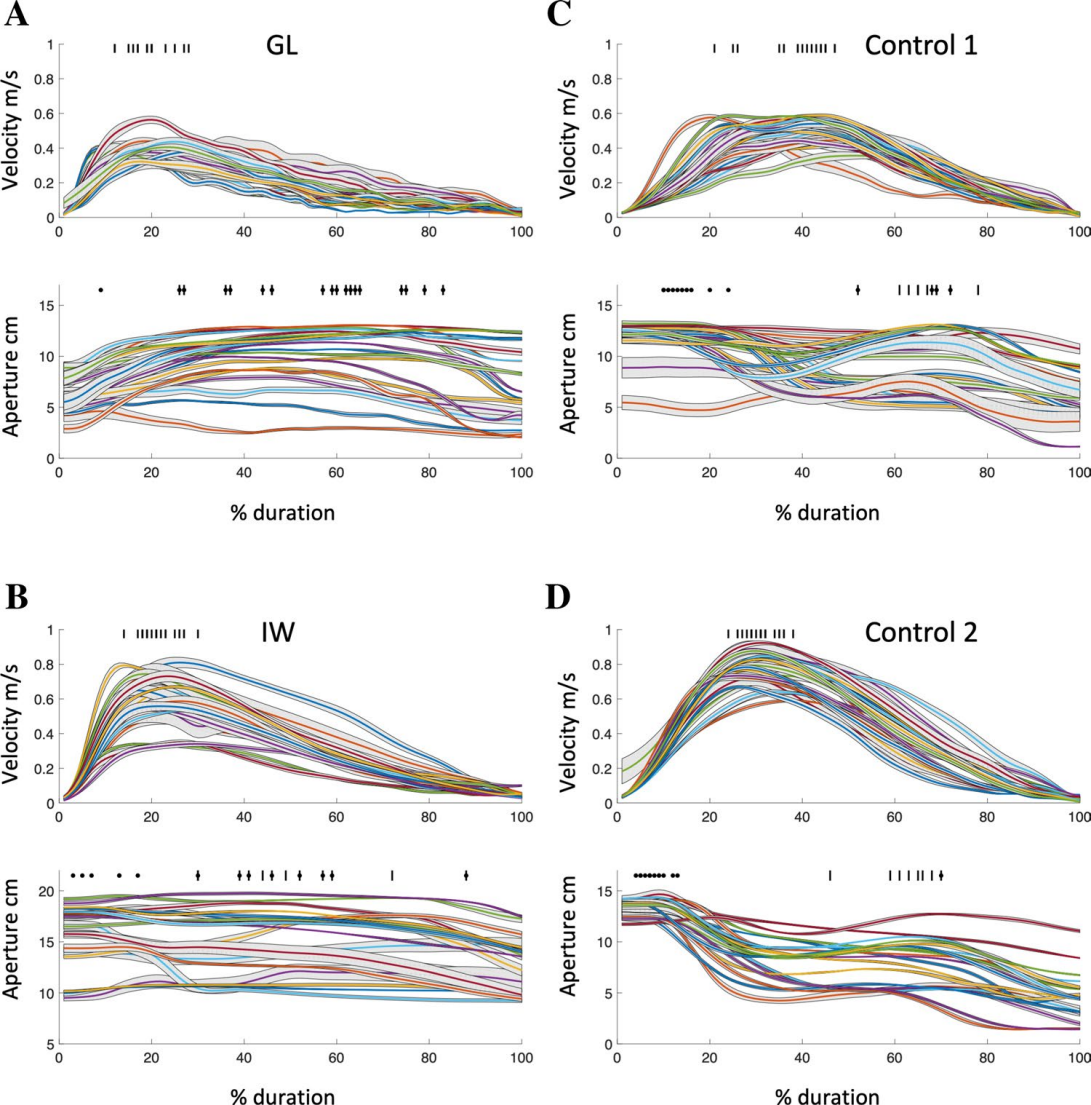
Average reach-to-grasp behaviour for each object. In each panel **a**–**d**, the upper graph shows the mean speed profile for each object (m/s; mean ± 0.5 SEM across five trials); the tick marks indicate the moment of maximum speed. The lower graphs show the mean grip aperture between thumb and index finger (cm; mean ± 0.5 SEM). The black dots indicate the moment of maximum mean aperture; the tick marks indicate the maximum aperture that follows the maximum speed. The horizontal axis represents % reach duration (see “Methods”). Controls 1 and 2 are the same participants as in Fig. 1

The differences between these deafferented and control participants in the kinematics of the reach-to-grasp phase are further emphasised in Fig. 2, where we plot the average speed and aperture profiles for all objects, after resampling the time-course data to 100 samples per trial. There was a range of apertures adopted during reach-to-grasp, as appropriate to the range of object sizes. However, GL and IW appear to have a “plateau” in grip aperture from about 40 to 90% of the approach duration, during which the aperture is relatively stable. In contrast, for most of the control participants (of which Controls 1 and 2 are representative), grip aperture varied more, both between objects and across the reach-to-grasp duration, especially late in the reach action. Figure 2 (bottom panels) specifically shows that for the controls, moment of maximum aperture (represented with tick marks) was clustered at around 75% of the duration, but varied widely for GL and IW. Figure 2 also shows the mean speed of the hand as it approached the objects. Both GL and IW adopted an asymmetric speed profile with high initial speed and a slower final approach. Here, Controls 1 and 2 are less representative of the other controls, as they were selected on the basis of the similarity of their speed profiles to GL and IW, respectively. Overall, across objects, the moment of peak speed (vertical tick marks, Fig. 2, upper graphs) was significantly earlier for the two deafferented participants (average of 23.5% and 23.3% of the reach-to-grasp duration for GL and IW, respectively) compared with a control group average of 35.6% [mixed-model ANOVA, with factors of group and object, group: *F*(1,6) = 8.22, *p* = 0.029]. The factor object was not significant [*F*(18,108) = 0.5, *p* = 0.9]; nor was the object–group interaction [*F*(18,108) = 1.4, *p* = 0.13]. In summary, these behaviours (a greater normalised aperture, an extended period of steady aperture and a relatively longer final approach) may reflect a strategy chosen by the deafferented individuals to provide a greater safety margin as they are going to grasp the object.

**Fig. 3:**
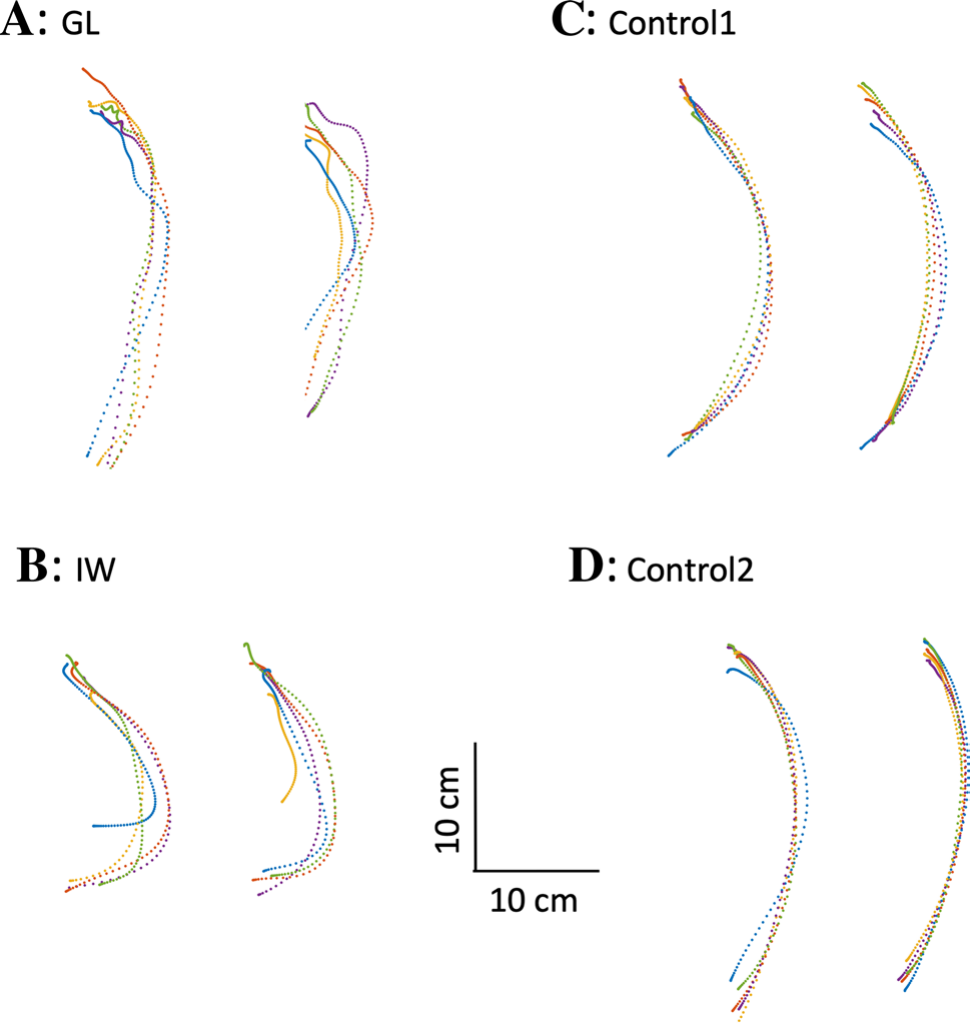
Reach-to-grasp paths. In each panel **a**–**d**, the graphs show a top–down view of the hand paths taken to reach the transparent plastic sheet (object 2, left graph, five trials) or the black opaque sheet (object 3, right graph), with the graphs rotated to approximately align the start and end vertically; the reach movements start at the bottom, and the object being grasped is at the top of the graphs. The data for IW, who is left-handed, have been mirror reversed. Individual trajectories have been spatially aligned using Procrustes analysis (see “Methods”). Controls 1 and 2 are the same participants as in Figs. 1 and 2

It was also apparent, especially for IW and WL (no data provided), that they reached for the objects with an abnormal hand path, with trajectories that were laterally curved, resulting in a rather side-on approach (shown for IW in Fig. 3; note this curvature was rightward for GL and the controls, and leftward for IW who is left-handed). The figure also shows the relative reproducibility across trials in control participants compared with the more variable reaches of IW and GL. However, to be documented properly, these spatial features of the approach path require more careful control of initial reach distance and position.

#### Hand postures during object lift

**Fig. 4:**
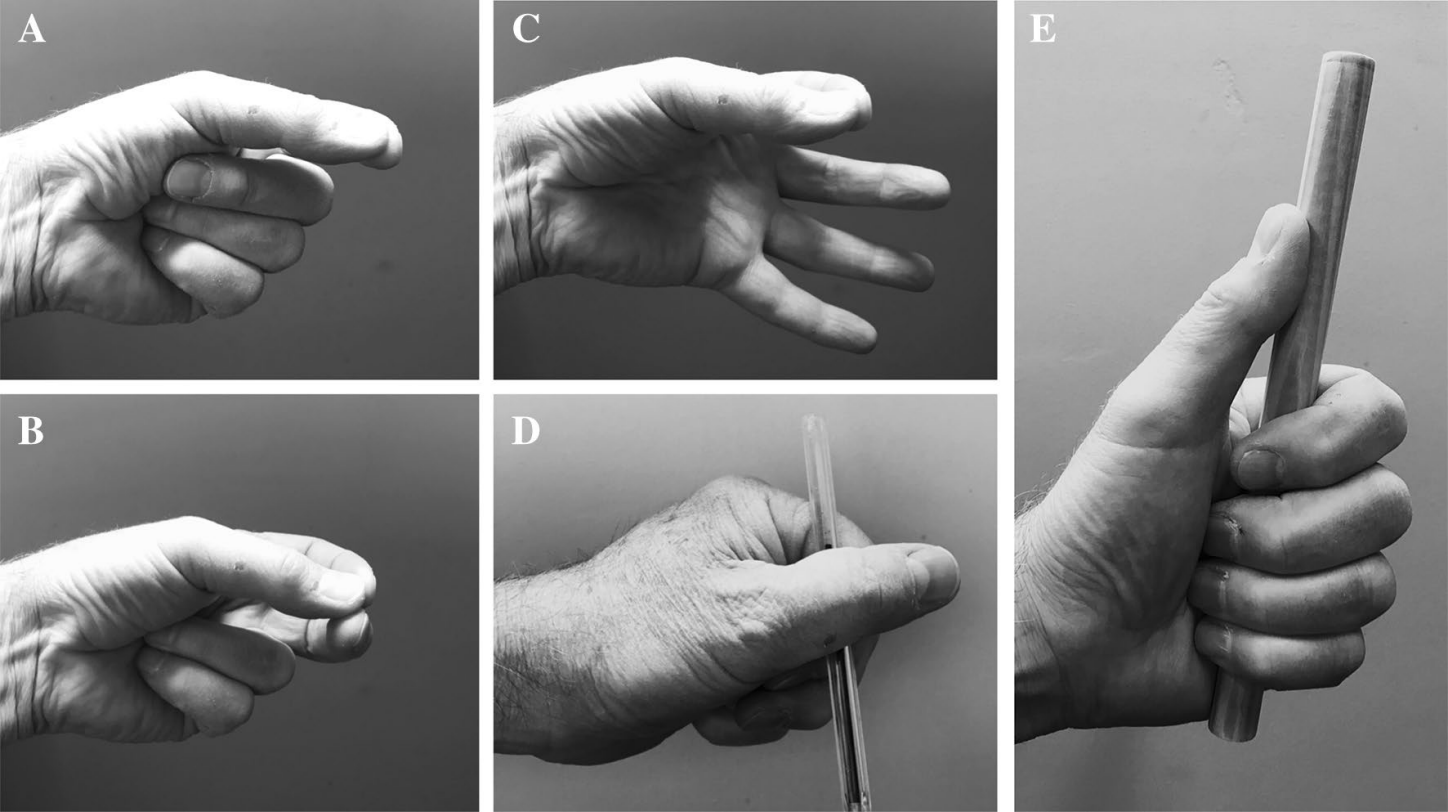
Example hand postures, from a model mimicking some of IW’s postures. **a** Two-digit precision grip, with other fingers flexed into palm; **b** three-digit version of **a**. **c** two finger grip with other fingers extended. **d** Cross-thumb pencil grasp. **e** An adducted thumb power grip; if the thumb is lifted off the object, this is known as a fixed hook (Feix et al. 2016)

In general, the deafferented participants avoided a precision grasp between just thumb and index fingers (Fig. 4a, c) and instead stabilised the held objects with multi-digit postures or between the thumb and the lateral side of the flexed index finger (Fig. 4d; a cross-thumb grasp; Bergmann 1990). For example, GL attempted to grasp a 1.5-cm-diameter cylinder (object 4) with a precision grip but the object slipped during the lift phase. On subsequent trials, she used a cross-thumb grasp. In addition, in holding the pencil as they do when writing, all three deafferented participants used a cross-thumb grasp. In contrast, the controls tended to adopt a “tripod” pose (Feix et al. 2016) with the pencil between the tips of thumb, index and middle fingers. Once grasped, the deafferented participants manipulated some objects (frequently the narrow cylinders) into a more stable hold: for example, IW and GL initially grasped the narrow cylinders with a tripod composed of two fingers and the thumb (like Fig. 4b, but with other fingers extended as in Fig. 4c) before changing to either a fixed hook or adducted thumb power grip (Fig. 4e; Feix et al. 2016).

**Fig. 5:**
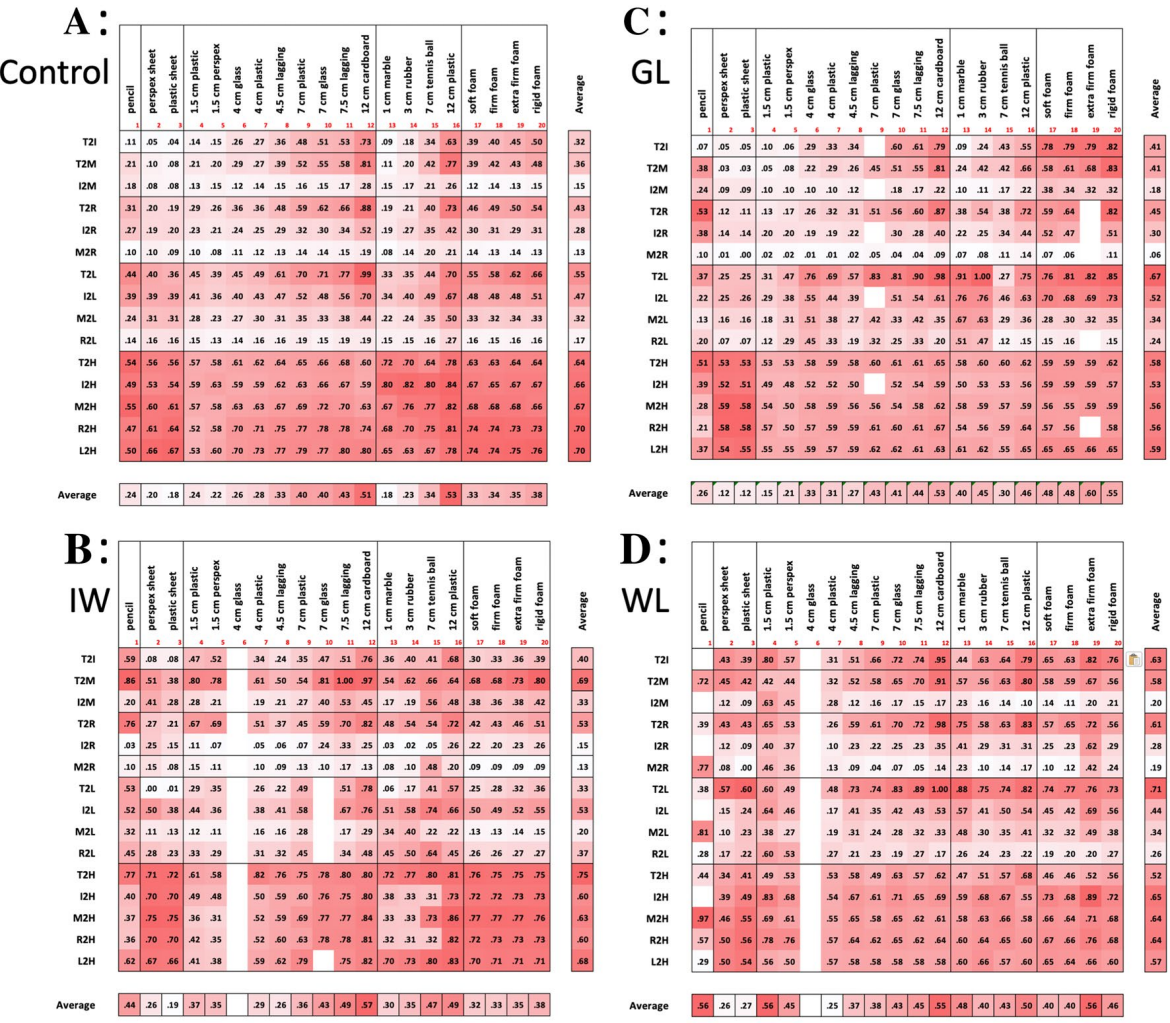
**a** Normalised finger–finger distances for each object for the control group. Each cell is the average across trials (*n* = 5) and control participants (*n* = 6); each row is one pair of markers: thumb, index, ring, middle and little fingers (T, I, M, R, L) and palm of the hand (H). Thus, the top row, for instance, is the average thumb-to-index finger distance (T2I). Averages across digit pairs and across objects are shown at the bottom and right, respectively. The other three panels (**b**–**d**) are from the individual deafferented participants GL, IW and WL, with each cell the average across the five trials. Objects have been clustered by shape (sheets, tubes, spheres and cubes) and ranked by size; the foam cuboids are ranked by compliance. The colour scale (deeper red for greater distance) is graded separately for each of the four panels **a–d**, and within each panel is separately graded for the five classes of objects (clusters of columns), and also for the averages (the separate row and column at the bottom and right of each panel). Empty cells reflect missing data

**Fig. 6:**
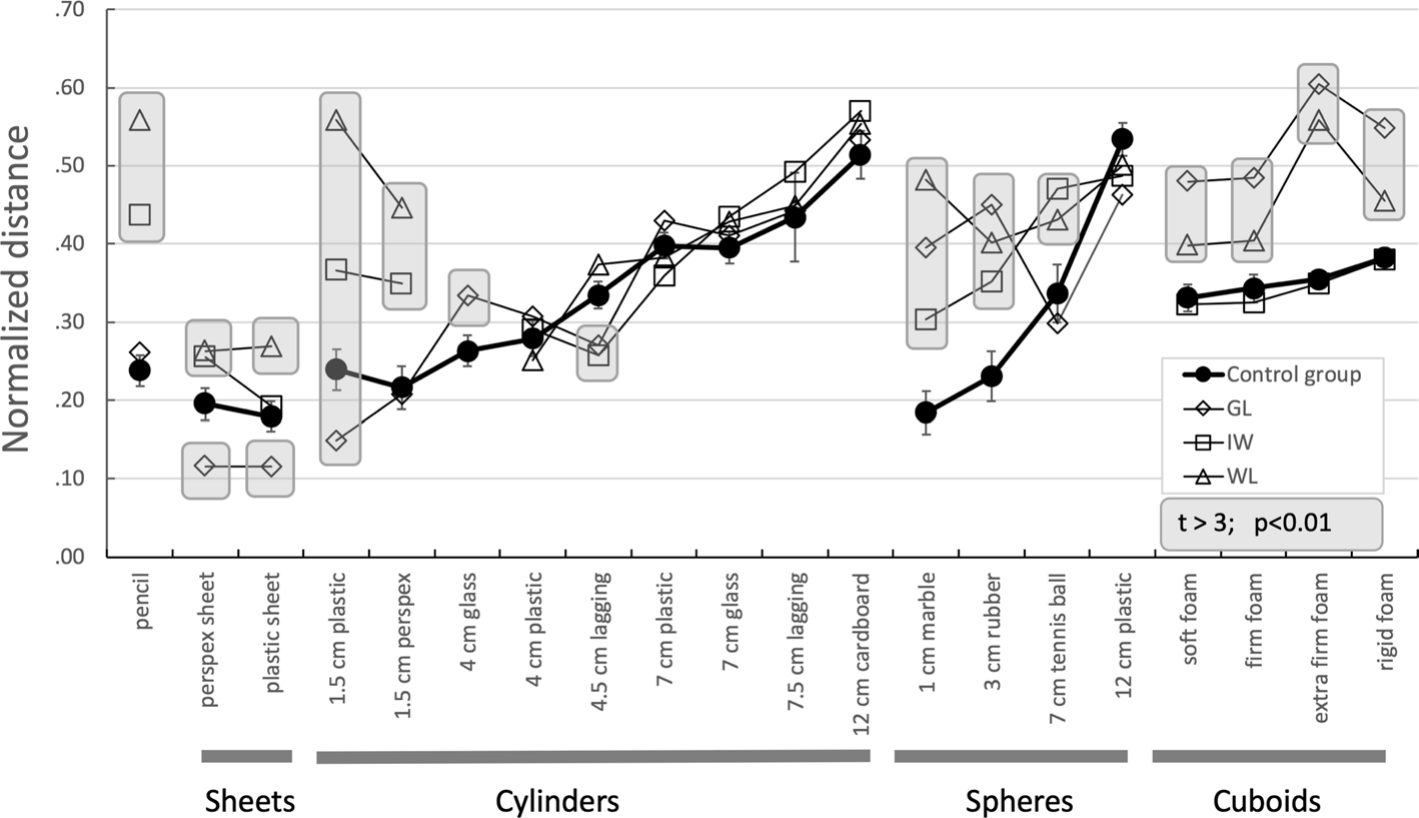
Average inter-digit distances (normalised for each participant to the range 0:1) for grasp of each object, for the control group (black filled dots, *n* = 6, ± 1 SD), and for three deafferented participants (where each data point corresponds to the average across the five trials for each object). Inter-digit distance was averaged across all digit–digit pairs, i.e. the column-wise average of data in the upper ten rows of the matrices in Fig. 5. The values for the three deafferented participants are highlighted with grey boxes when their *t* score > 3.0 (*p* < 0.01) compared to the control group

The deafferented participants also often used hand actions that appear to reduce their degrees of freedom. For example, when grasping the rigid sheets, they might flex all four fingers together as a unit (Santello and Soechting 2000) flexing at the metacarpophalangeal joint to act as a single, rigid ‘virtual’ finger (Arbib et al. 1985; MacKenzie and Iberall 1994). In addition, they might limit contact with the object to just three fingers, and keep the others clear. Hence, GL often used a posture with the lateral fingers (ring and little) fully extended; for example, when she grasped small cylinders or spheres with the medial three fingers, the other two (ring and little fingers) were fully extended. It was also noticeable that when she released objects, these lateral fingers were the first fingers to be extended. We also observed that when GL grasped the cubes, she did not use the index finger which was fully extended. In contrast, IW was often observed to flex the ring and little fingers into the palm of his hand (Fig. 4a, b), again potentially simplifying the control problem by eliminating movement of redundant effectors. These behaviours are further quantified below (Figs. 5, 6).

#### *Inter*-*digit distance matrices*

We calculated a distance matrix for each participant, from the mean normalised distances between all possible pairs of the 6 markers, averaged across trials per object (Fig. 5). For the control group, we also averaged the normalised matrix across participants. As expected, there was a systematic scaling of digit-to-digit distances as objects increase in size within each class (for example, for the cylinders, objects 4–12, Fig. 5a). One can also see subtle scaling of the distances between the five fingers and the palm (Fig. 5a, bottom five rows of the matrix) especially for the smallest objects (e.g. objects 1, 4, 5 and 13). This reflects the differential configurations of the fingers such that the fingertips are closer to the palm for some objects than others. Figure 5b–d shows the corresponding matrices for the three deafferented participants: here, the scaling of distances is less ordered. The variance between the deafferented participants appears high compared to the control group (see the SEM bars for group data in Fig. 6, compared to the three deafferented participants). To assess this, given the small sample of deafferented participants, we compared the variance of the control group (*n* = 6) with the combined control and deafferented group (*n* = 9). If the deafferented participants were representative of the controls, the combined group should have the same variance. In fact, for almost all finger–finger distances the variance increased [grand average *F*(5,8) = 3.9, *p* = 0.05]; and for every object, the average of the *F* ratios was greater than 1.3. This suggests that variability between the deafferented participants was higher than expected from the control group.

Figure 6 shows the average of the digit-to-digit distances (i.e. the column-wise average of the data in the top ten rows of the matrices in Fig. 5), for the control group and for each of the three deafferented participants. The monotonically increasing relationship between object size and average digit-to-digit distance is clear for the controls’ data within each set of cylinders, spheres, and cuboids but is considerably disordered for the deafferented participants. The greatest differences from the control group are seen for deafferented participants IW and WL when grasping the smaller items (small diameter cylinders and small spheres), and in WL for the pencil (see left and central sections of Fig. 5). Their increased inter-finger distance reflects the tendency to fully extend one or more fingers for these small objects which cannot be reliably grasped from a flat surface by simple thumb–index finger opposition, unlike some of the medium-sized objects.

#### Transparency

For a subset of objects, we could compare transparent and opaque materials (objects 2/3, 4/5, 7/8 and 9/10). For the inter-digit distance, a three-way repeated-measures ANOVA with factors of object (sheet; 1.5-cm-, 4-cm- and 7-cm-diameter tubes), material (transparent vs opaque) and group revealed significant differences between objects [*F*(3,21) = 56, *p* < 0.0001], but no significant main effect of material [*F*(1,7) = 0.07, p ≥ 0.7] or group [*F*(1,7) = 3.14, *p* > 0.2], nor any significant interactions.

#### Density

We examined the possibility of a positive relationship between inter-finger distance and the density of the four foam blocks, on the assumption that compression of the more compliant blocks would lead to reduced finger distances. Such relationship was clear in both the controls and deafferented participants (with a rising value for average inter-digit distance with density, see right panel of Fig. 6), and an RM-ANOVA across both participant groups showed a significant effect of the density factor [*F*(1.75,12.2) = 15.8, *p* = 0.001, Greenhouse–Geisser adjusted because of violation of sphericity] as well as a strong linear contrast effect [*F*(1,7) = 42, *p* < 0.0001]. There was, however, also a significant interaction between the group (control vs deafferented) and density [*F*(1.75,12,24) = 6.07, *p* = 0.017], and performing linear regression of inter-finger distance against density showed the regression coefficient was statistically significant only for the controls (*t* = 2.2, *p* = 0.037) and not for the deafferented participants (*t* = 1.2, *p* = 0.27).

#### Average hand patterns

**Fig. 7:**
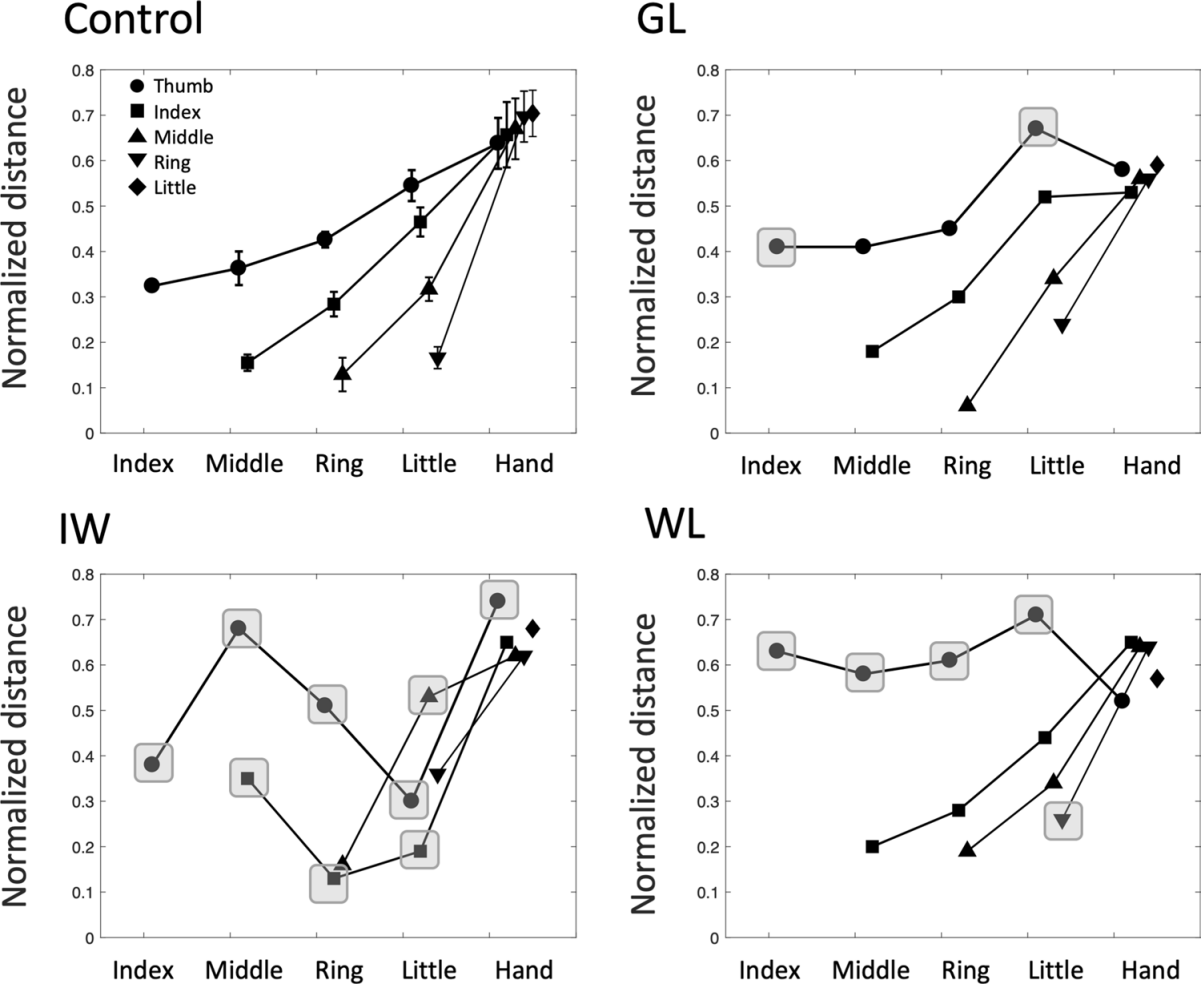
Average inter-digit distances for the control group (mean ± 1 SEM, *n* = 6) and the three deafferented participants. Each data point is the normalised distance between a pair of digits, averaged across all the objects, as shown in the right-most column of each panel in Fig. 5. Each line represents the distance from one finger (see legend in the upper-left panel) to the other fingers or to the palm of the hand (target digit on the horizontal axis). Hence, the uppermost line (with circle symbols) is the distance from the thumb to other digits, and the left-most data point is the average thumb–index finger distance. The next line is for the index finger (squares), and so on. The right-most singular data point (diamond symbol) is the average little finger to palm distance. The datasets have been slightly offset horizontally for clarity. Individual data points for the three deafferented participants are highlighted when significantly different from the control group (*t* score > 3.0; *p* < 0.01)

Turning next to examine systematic differences in holding postures, Fig. 7 shows the normalised digit–digit distance between all possible pairs, averaged across all objects (i.e. the average across all columns, for the first ten rows of the matrices in Fig. 4). Here, the average distance of the thumb to other digits is generally greater for the deafferented participants than for the controls (see the grey highlights in Fig. 7). This may be explained by the tendency of the deafferented participants to splay the hand wider than normal and to extend the ring and little fingers. IW adopts either this pattern, with ring and little fingers fully extended, or alternatively, he folds the ring and middle fingers into the palm of his hand; this leads to differences in average thumb–ring and thumb–little finger distances shown in Fig. 7.

#### Variability of hand posture

**Fig. 8:**
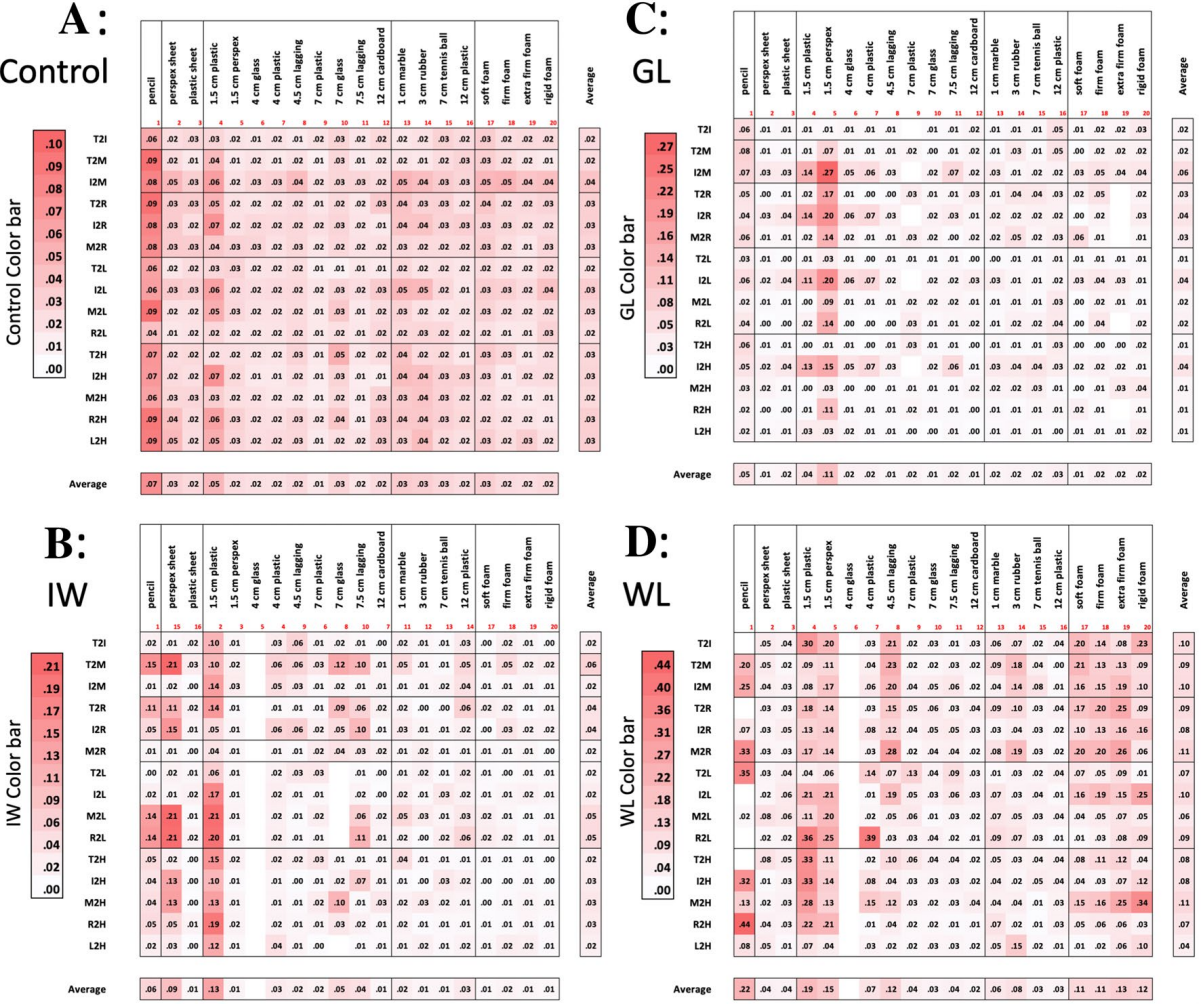
Trial-to-trial variability of the normalised finger–finger distances, across objects. For the control group (top left), each cell is the average across participants (*n* = 6) of the standard deviation estimated from the five trials per object; the format is the same as in Fig. 5. The other three panels are from the individual deafferented participants (IW, GL and WL), with each cell showing the standard deviation of the distances across the five trials. Colour bars give the scale for each panel; note the range for the controls (**a**) is 23–48% smaller than all others

Figure 8 shows the variability of hand postures across the five trials with each object, with each cell showing the standard deviation of the data shown in Fig. 5. For the control group (upper-left panel), the data are the group averages of the standard deviations for each individual. Control participants had generally very low variance, and there appears to be little structure in the variance across classes of objects. In comparison, the three deafferented participants showed higher within-object variability than the controls (note change in colour scale), most pronounced for the smaller cylindrical objects: the pencil and tubes of 1.5-cm diameter; WL also showed higher variability for the cuboids. A mixed ANOVA showed the group effect was not significant [*F*(1,7) = 2.8, *p* = 0.17] while the object factor was significant [*F*(18,126) = 5.65, *p* < 0.001] and the group–object interaction was also significant [*F*(18,126) = 1.9, *p* = 0.021].

#### Patterns of hand posture

**Fig. 9:**
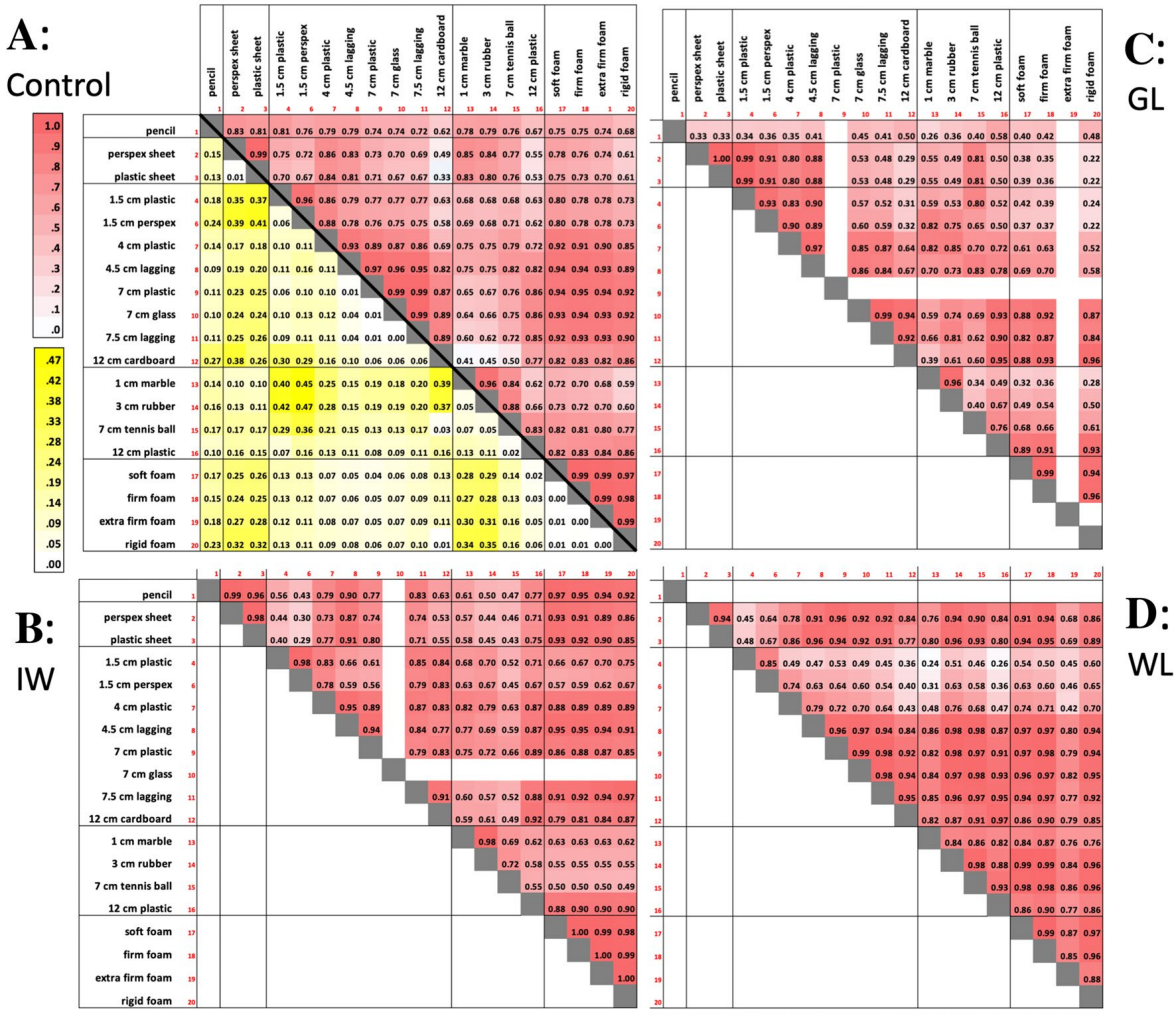
Assessment of the correlation of hand postures between pairs of objects. In each panel **a**–**d**, we display above the black diagonal the correlation between hand postures for all pairs of objects, with posture quantified by the 15-element finger-to-finger distance vector (Fig. 5). For the controls (panel **a**), each of these cells indicate the group average of the individual correlations (*n* = 6). Below the diagonal of panel **a**, we show the standard deviation of these correlations across the group. Hence, highly correlated postures are in darker red, above the diagonal, and those with maximum variability across the control group are in bright yellow. The remaining panels (**b**–**d**) are individual data from the three deafferented participants, IW, GL and WL, respectively. Blocks with less than three good trials were excluded, leading to missing correlations

To compare hand postures across objects, we performed correlations of the sets of 15 finger-to-finger distances (i.e. the full columns in Fig. 5) for all possible pair-wise combinations of objects (Fig. 9, upper right of each panel). Thus, each hand posture was defined by the 15-element distance vector averaged across trials, and high correlations between these vectors imply similar hand postures for pairs of objects. For example, for the controls, there was high correlation between the grasps used for objects 17–20, the four cuboids (as indicated by the dark red cells in Fig. 9a, at the right side of the matrix, just above the diagonal). There was also a high correlation between these hand postures and those used for the larger cylinders (objects 8–11, dark red cells on the right margin of Fig. 9a). We also show the standard deviation across the control group in yellow colours below the diagonal. The least variability in the correlations was seen for the cuboids, and for the largest cylinders. In contrast, and as might be expected, there was low pair-wise correlation between the hand postures used for large tubes and small spheres (the former requiring a maximally extended power grasp and the latter a precision grip), but also higher variability in these correlations across the control group as they used more idiosyncratic postures to hold small items. Thus, there was a low mean correlation between the hand posture used to grasp the 12-cm tube (object 12) and the hand postures used for the smaller spheres (the row of pale cells at the centre of the matrix, above the line), and high across-group variability in the correlations (the brighter yellow cells, below the diagonal). The picture for the deafferented participants was more varied. GL showed relatively low correlations between many object pairs, hence grasp postures varied (pale red cells in Fig. 9c). In contrast, IW tended to have more correlated sets of hand postures (for example, adopting similar postures for sheets, medium cylinders and the foam cuboids, Fig. 9b), but he showed low correlation between the hand postures for small spheres and other objects. WL also showed low correlation between her hand postures for smaller cylinders and other objects. Finally, there were relatively high correlations between hand postures, in GL, IW and WL, for the pairs of objects with different transparency (e.g. for object pairs 2 and 3; 4 and 6, 7 and 8; Fig. 9).

## Discussion

The three deafferented participants that we tested have lived with a profound sensory neuronopathy for many years and have impaired manual abilities. Their ability to grasp and lift everyday objects hides the significant control problems that they have overcome.

### The role of haptic feedback in reach-to-grasp movements

The deafferented participants appeared to take longer to reach towards objects, with a longer deceleration phase relative to movement duration than controls. This is consistent with previous reports on individuals with hypoesthesia due to peripheral (Gentilucci et al. 1994; Hoellinger et al. 2017) and central causes (Jeannerod et al. 1984, 1994). As discussed in those papers, a longer movement duration likely reflects greater task difficulty and a greater need to use visual feedback.

They also held a large grip aperture over a longer proportion of the reach duration, also consistent with the idea of increasing the margin of safety during the approach. Jeannerod et al. (1984, 1994) also reported abnormal extension of the fingers during the reach-to-grasp phase for individuals with hypoaesthesia due to central lesions, while Whitwell and Goodale (2009) showed that when visual feedback is not available, grip aperture is increased. Gentilucci et al. (1994) also reported high trial-to-trial variability of maximal finger–thumb aperture when a peripherally deafferented participant approached objects of different sizes. This is analogous to the high variability in movement trajectories that has been reported in these same deafferented participants when pointing or moving a robotic manipulandum to visual targets (Gordon et al. 1995; Sarlegna et al. 2010b; Miall et al. 2018).

It was also apparent, especially for participants IW and WL, that they approached the objects with an abnormal hand path that had higher lateral curvature, consistent with the work of Jeannerod et al. (1984). We speculate that this allowed better visual monitoring of the hand and fingers for as long as possible. However, we had not designed our experiment to carefully control the reach-to-grasp phase of the actions, intending to keep the grasp and lift of each object as natural and unconstrained as possible. Also, we only captured the full reach trajectory for GL and IW. Hence, we will need to return to this issue in later experiments.

### Role of haptic feedback during object lifting

The pencil, small cylinders and spheres appeared to be more difficult for the deafferented participants to lift off the table than larger objects. The difficulty in grasping and lifting the pencil was that the task required not only the pick-up of a narrow cylindrical object from the table surface, but also the challenge of then manoeuvring it into a functional writing pose. This manipulation phase may be particularly challenging, as it potentially requires simultaneous control of all five digits. The other objects tested had no obvious functional demands on how they were grasped, and so did not require manipulation once lifted. A finger–thumb opposition may be one strategy to simplify control of the digits, especially for small objects, but it provides unstable grip in the absence of haptic signals (Augurelle et al. 2003; Johansson and Cole 1992; Monzee et al. 2003; Westling and Johansson 1984). The deafferented individuals face the dilemma that a precision grip does not provide sufficient stability but using additional fingers results in more complex control problems (Sainburg et al. 1993). Alternatively, the thumb may be opposed against all four fingers that act as a single broad ‘virtual finger’, but adequate opposition between the finger pads is difficult to achieve with straight fingers: this posture may be sufficient for grasping the plastic sheet, for example, but cannot be used for picking up small items from a flat surface.

For medium-sized objects (e.g. cylinders and foam cuboids), the exact placement of the fingers on the object may be unimportant as long as thumb–finger opposition is achieved. The deafferented participants often displayed more trial-to-trial variation than normal: this may reflect the use of null-manifold optimal control (Todorov and Jordan 2002; Shim et al. 2003) where variability in placement of some fingers is tolerated, or even exaggerated, as long as it allows them to achieve good control in other task-critical dimensions. However, with large objects, variability of hand postures may drop because all four fingers tend to operate as a linked unit, opening to the extremes of their range, and are jointly opposed by the thumb.

In line with the idea that unifying principles which reduce degrees of freedom underlie multi-digit control, IW has reported that he attempts to “simplify” the hand postures he uses. He reported that he does this by tucking his ring and little fingers into the palm, or by extending middle, ring and little fingers, thus restricting active control to a pinch grip between thumb and index finger, or a tripod grip with thumb, index and middle finger. This strategy was in fact evident in all three deafferented participants, for particular objects, indexed by the higher average thumb or index to little finger distances (T2I or I2L, Fig. 7).

### Role of vision during object holding

IW has reported that controlling his fingers when they are occluded behind an opaque object is more difficult than when using transparent objects. Based on the volume of work highlighting the role of visual feedback in deafferented individuals (Blouin et al. 1993; Ghez et al. 1995; Ingram et al. 2000), we predicted this might lead to different grasp postures for transparent and opaque sheets and cylinders. But in fact the hand postures during object lifting did not statistically differ and we observed relatively high correlations between hand postures, for the pairs of objects with different transparency (e.g. for the Perspex vs plastic sheet, or Perspex vs plastic cylinders, Fig. 9). On questioning, after the task, one can also observe finger contact through a transparent object, and potentially even observe the change in skin colour as the fingers press against the object. GL reported that she does not consciously use the colour change of the fingertip to control grip force. Hence, if hand postures were influenced by the visual properties, it was not in a systematic fashion either between object pairs or across the deafferented participants. Further studies are, thus, needed to determine which exact visual signals and mechanisms are used to control finger movements and forces.

### Compliance, haptic feedback, perception and action

We had predicted that the supranormal grip forces presumably applied by deafferented participants to objects might result in compression of highly compliant surfaces, and hence reduced finger–finger distances. In fact, the opposite was found for the foam cubes, and there was greater inter-finger distance than for the controls. This appears to be consistent with the idea that deafferented participants greatly rely on vision for movement control (Blouin et al. 1993; Ghez et al. 1995; Sarlegna et al. 2010b; Miall et al. 2018), as they might visually monitor the finger configurations and the object shape in order to finely control contact forces, as suggested by other work (Jenmalm and Johansson 1997). Indeed, deafferented participants can maintain steady grip forces when provided with visual feedback and can maintain grip and load force control when observing their lifting actions (Hermsdörfer et al. 2008). As a follow-up to the grasp and lift task, we asked IW to rank the compliance of the four foam blocks and he was able to do so, with vision, by judging the compression of the objects as he pressed down on each of them in turn. Thus, when lifting the most compliant objects, IW, GL and WL all appeared to use minimal grip forces and only marginally compressed the foam, guided by visual feedback. Overall, our findings support the idea that visual signals can contribute to haptic interactions (Fleury et al. 1995; Lécuyer 2009; Sarlegna et al. 2010a; Cuadra et al. 2019).

### Individual differences

While we have only tested and reported on three deafferented participants, studies of these very rare cases are few, and so it is tempting to try to understand the differences in their performance, and in their strategies when grasping and lifting objects. Comparing across the 20 objects, GL showed low correlations between many grasp postures (pale red cells, Fig. 9c). Moreover, Fig. 8c suggests she has lower variability across trials than IW or WL, although she was variable in placing her index finger, with higher mean variability in I2M and I2R and I2L distances for the small cylinders, for example. In contrast, IW tended to have more clearly correlated sets of postures (adopting similar postures when grasping sheets, medium cylinders and the foam cubes, as shown by darker red blocks in Fig. 9b), but he also showed higher variability across trials with any one object (Fig. 8b). We suggest this difference between IW and GL reflects their different levels of cognitive control: IW plans, attempts high levels of control, and is frustrated by his mistakes. He states that he must concentrate on each action, and prefers to take time to prepare every one, perhaps leading to uncorrelated trial-to-trial fluctuations. GL appears less controlled, often faster to initiate each trial, and appears less concerned by errors when participating in scientific experiments (see also Miall et al. 2018). WL also showed low correlation between her postures, especially for smaller tubes and other objects, and the highest variability. However, WL is more ataxic than either GL or IW, and some of her variability may reflect poor control of the actions, rather than intended postural differences. Finally, in both IW and WL’s cases, we may also interpret low correlation between object postures as a reflection of strategies adopted object-by-object, to simplify control, for instance by reducing the grasp to a tripod posture, with ring and little fingers either extended or fully flexed. So, as seen in some other tests (e.g. Miall et al. 2018; Renault et al. 2018), these deafferented participants are not a homologous group and may use different strategies to each other, and even between different objects.

## Conclusion

Overall, these findings suggest that when deprived of peripheral sensory information, new control solutions are found. We found that they often adopted postures that reduced the degrees of freedom of the hand, either folding the ring and little fingers into the palm, or extending them, so that only the thumb, index and middle fingers were active in the grasp. Another simplification was to flex the four fingers together at the proximal MCP joint, as one unit, while also reducing the curvature of this single “virtual finger”. However, they did not often use a simple pincer opposition between thumb and index finger, unlike controls, needing more stable support to avoid object slip. We found no evidence that the postures differed when the objects were transparent or opaque. However, they appear to use visual control of pressure on compliant objects, compressing them less than controls. Despite these presumably adaptive changes, individuals with sensory neuropathy still present impairments in everyday manual abilities. There were idiosyncratic differences between the three deafferented participants, that may reflect their differing levels of ataxia, and potentially differences in cognitive control of action. There were increases in variability across trials with the same object, and also changes in the pattern of hand postures between objects that implied less systematic, or less automatic, control of the hand to conform to the shape to be held. This control of hand movements during everyday physical and social interaction with the environment is precisely the task that spinal-cord injury patients wish to be able to perform again (Snoek et al. 2004). The present study also highlights for prosthetic or brain–machine devices, the need to restore tactile and proprioceptive signals (Armenta Salas et al. 2018; D’Anna et al. 2017; Saal et al. 2017).
